# Proceedings of Various Medical Societies

**Published:** 1850-09

**Authors:** 


### ARTICLE IV.

*Proceedings of the Medical Society of the State of Pennsylvania, at its Annual Session, held in Philadelphia, April,* 1850; pp. 55; octavo.

*Minutes of the Proceedings of the South Carolina Medical Association, at its Annual Meetings, February,* 1849--50. Charleston, S. C.: Walker & James; 1850.

*Proceedings of the First Annual Meeting of the Indiana State Medical Society, held in the City of Indianapolis, May,* 1850. Indianapolis: Elder & Harkness; 1850.

*Sixty-third Annual Report of the Regents of the University of the State of New York, made to the Legislature, March 1st,* 1850; pp. 379, octavo.

*Transactions of the Medical Association of Southern Central New York, at the Annual Meeting held at Elmira, June,* 1850; pp. 80; octavo.

One of the greatest advantages resulting from the formation of the National Medical Association, is the impulse thereby given to the organization of the profession throughout the whole country. Since that body has begun to hold its annual essions, many State Medical Societies have been organized; and the natural operation of these is to induce the profession to form local auxiliary Associations, thus giving impetus to the spirit of reform and inquiry. The medical profession has complained, loudly and justly, of many disadvantages under which it has labored, and of various indignities which have been inflicted upon it; but until lately there has appeared but little rational hope for relief. The old fable of the bundle of sticks, or the motto "that in union there is strength," has not until lately been appreciated or acted upon; and the consequence has been, that although there were many energetic and spirited efforts put forth in various quarters for the advancement of the profession, a lack of concert of action rendered them comparatively inefficient. But a brighter day is now dawning. A thorough organization, enabling the profession to act as one man, cannot fail to produce important results. A body of men so numerous and respectable as the medical profession of this country, cannot fail to secure attention and respect, if they are united in demanding it.

We ask no political immunities nor protection, and we demand that no petty demagogue nor political busy body shall insult us with silly enactments, merely for the purpose of ministering to the stupid prejudice of some half-dozen blockheads, whose votes he covets. We can take care of ourselves, and are willing to trust the good sense of the people to discern between the man of merit and the impudent pretender; for if public opinion sometimes gets wrong, legislative opinion, which is only its echo, is quite as likely to go astray. Let the profession become thoroughly organized, and let each member strive to improve the profession by improving himself, and we have no fear that we shall be superseded in the confidence of community by empirics and pretenders. But we must not lose sight of the fact that people judge of merit abstractly. They do not enquire where a physician obtained his diploma, nor whether he has got one; but is he a *good doc-*

*tor?* does he cure his patients? And herein they are right. If we cannot cure more than the quacks, we are no better than they; and if we evince superior skill, it cannot fail to appear ultimately; for although deception may flourish for a season, it must, in the nature of things, be sooner or later unmasked. The following resolution of the Indiana State Medical Society, though not very clearly expressed, embodies the true doctrine on this subject:—

WHEREAS, The protecting arm of the law has been withdrawn, in the State of Indiana, from the Medical Profession; And whereas, it is expedient that a broad line of distinction between Scientific Medicine, and the various forms of empiricism in vogue in our country should be plainly marked, and seen. Therefore,

*Resolved*, That this object is mainly attainable by the influence to be exerted by individual members, and as a consequence of elevating the standard of their own professional acquirements.

We are glad to see that the interest of the Indiana physicians in the success and usefulness of their State organization is increasing; and it is almost superfluous to say that our warmest wishes for the continued prosperity of the Society are cordially tendered.

We notice that it is in contemplation to hold the annual sessions of the Society at various points in the State. We think this will have a good effect, serving, as it will, to interest the profession of each section of the State, in the meetings of the Society. The following are the officers for the ensuing year:—

*President.*—Dr. A. CLAPP.

*Vice-Presidents.*—Drs. WM. LOMAX, R. CURRAN, W. DAVIDSON.

*Recording Secretaries.*—Drs. J. S. Bobbs, A. M. Hunt.

*Corresponding Secretary.*—Dr. T. Bullard.

*Treasurer.*—Dr. D. Funkhouser.

*Librarian.*—Dr. P. H. Jameson.

*Delegates to the National Medical Association.*---Drs. R. C. Hamill, L. Dunlap, R. Farquar, Jno. Sloan, T. Ryan.

The report of the proceedings is not as full as it might be; as we see no notice of the address delivered before the Society, by our editorial *confrere*, descriptive of a new Obstetrical Instrument invented by him.

Appended to the proceedings is an able and interesting address by the President, Dr. Cornet.

In the proceedings of the State Medical Society of South Carolina, we notice several very interesting features. It is pleasant to observe the very proper and cordial friendship existing between the Society and the Governor, and the high appreciation of the services rendered by the profession to community. We are gratified, also, at the strenuous efforts made to complete the indigenous medical botany of the State, and the decided stand taken in reference to the subject of adulterated and spurious drugs. We confess, however, that following resolutions are not exactly to our mind :—

*Resolved*, That the members of the South Carolina Medical Association will continue to offer their services gratuitously to all clergymen and their families, when the clergyman is known to be a friend of the medical profession.

*Resolved*, That no clergyman shall receive our services gratuitously who advocates and recommends the use of secret and patented medicines, either publicly in the newspapers or privately to his own parishioners.

We believe that a clergyman is as much bound as other men to pay for medical services ; and there is no reason why his physician's fees should not be put on the same footing as his butcher's and baker's bill, unless he be in straitened circumstances. Nor do we believe that clergymen generally expect such services gratuitously; though we are pained to say that individual instances have come under our notice, where medical men have rendered the most arduous and important services to clergymen and their families without receiving so much as a single expression of gratitude in return; and this in cases, too, where the income of the clergyman was much greater than that of the physician. As to discriminating between those who recommend quack medicines and quack doctors, and those who are more enlightened, we do not think it amounts to anything. While we evince our respect to the latter class, we should be lenient to the former, and say to them kindly, "I wot, brethren, that through ignorance ye did it." It seems to us such a course is much more likely to reclaim the erring, and is inconceivably more useful than to exclude them from the pale of our good will and friendship, thus driving them, as it were, into the communion of quackery.

With regard to the other Associations mentioned in the caption of this article, we have space only to say that they appear to be in a prosperous condition, and continue to evince a determination to continue active in the cause of professional improvement. M.

